# A comparative study of microbial diversity and community structure in marine sediments using poly(A) tailing and reverse transcription-PCR

**DOI:** 10.3389/fmicb.2013.00160

**Published:** 2013-06-18

**Authors:** Tatsuhiko Hoshino, Fumio Inagaki

**Affiliations:** ^1^Geomicrobiology Group, Kochi Institute for Core Sample Research, Japan Agency for Marine-Earth Science and TechnologyNankoku, Kochi, Japan; ^2^Geobio-Engineering and Technology Group, Submarine Resources Research Project, Japan Agency for Marine-Earth Science and TechnologyNankoku, Kochi, Japan

**Keywords:** 16S rRNA, less biased diversity analysis, hydrothermal sediment

## Abstract

To obtain a better understanding of metabolically active microbial communities, we tested a molecular ecological approach using poly(A) tailing of environmental 16S rRNA, followed by full-length complementary DNA (cDNA) synthesis and sequencing to eliminate potential biases caused by mismatching of polymerase chain reaction (PCR) primer sequences. The RNA pool tested was extracted from marine sediments of the Yonaguni Knoll IV hydrothermal field in the southern Okinawa Trough. The sequences obtained using the poly(A) tailing method were compared statistically and phylogenetically with those obtained using conventional reverse transcription-PCR (RT-PCR) with published domain-specific primers. Both methods indicated that Deltaproteobacteria are predominant in sediment (>85% of the total sequence read). The poly(A) tailing method indicated that Desulfobacterales were the predominant Deltaproteobacteria, while most of the sequences in libraries constructed using RT-PCR were derived from Desulfuromonadales. This discrepancy may have been due to low coverage of Desulfobacterales by the primers used. A comparison of library diversity indices indicated that the poly(A) tailing method retrieves more phylogenetically diverse sequences from the environment. The four archaeal 16S rRNA sequences that were obtained using the poly(A) tailing method formed deeply branching lineages that were related to *Candidatus* “Parvarchaeum” and the ancient archaeal group. These results clearly demonstrate that poly(A) tailing followed by cDNA sequencing is a powerful and less biased molecular ecological approach for the study of metabolically active microbial communities.

## INTRODUCTION

Numerous studies on natural microbial communities from a variety of environments have been undertaken using 16S rRNA gene sequencing mediated by polymerase chain reaction (PCR) with oligonucleotide primers. In the past decade, high-throughput next-generation sequencing (NGS) technologies have facilitated the identification of a diverse array of organisms that are rare in terms of biomass and could not be examined using previous molecular assays such as Sanger sequencing analysis of clone libraries (Sogin et al., 2006;Webster et al., 2010). Despite the fact that the latest NGS technologies enable reading only of relatively short sequence fragments (~500 bp), these so-called “deep sequencing” methods are still powerful tools that ultimately may enable researchers to obtain a more holistic understanding of microbial communities in their natural environments (Fuhrman, 2009). Considering the current limitations of NGS technology, full-length 16S rRNA gene sequences are better suited to downstream analytical methods such as fluorescence *in situ* hybridization.

The original designs of most of the PCR primers used for the analysis of 16S rRNA genes were based on known sequences deposited in public databases. Researchers have since cautioned that these primer sequences contain mismatches with respect to environmental 16S rRNA genes (Baker et al., 2003,^2006^;Teske and Sørensen, 2008), which may lead to considerable bias in interpreting results (Hong et al., 2009). In addition, primer sequence mismatches may have a negative impact on the amplification efficiency of PCR analyses (Acinas et al., 2005;Sipos et al., 2007;Bru et al., 2008). Regardless of the presence of sequence mismatches, the use of PCR with primers may introduce biases associated with the next base adjacent to annealed oligonucleotide primers (Ben-Dov et al., 2012).

One way to avoid these bias issues is to employ PCR-independent metagenomic approaches. For example, a complete 16S rRNA gene sequence can be obtained by analyzing the sequences of genomes or large genome fragments, providing taxonomic information along with information on other functional genes. However, metagenomic approaches may not be well-suited to focused studies of microbial diversity and community structure that involve a large number of samples. In fact, it has been reported that only a small portion of inserts in fosmid libraries contain 16S rRNA genes (Vergin et al., 1998;Takami et al., 2012).

Another method that can avoid the possibility of bias caused by primer mismatching is the addition of a poly(A) tail to the 3′ end of fractionated 16S rRNA prior to synthesis of the full-length complementary DNA (cDNA;Botero et al., 2005). Since the technique does not involve the use of published primers, we anticipate that this method will enable recovery of full-length environmental 16S rRNAs, potentially illuminating as yet unknown microbial community constituents that have otherwise been difficult or impossible to detect using conventional PCR-dependent molecular approaches (Inagaki et al., 2002). In the present study, we tested this hypothesis using poly(A) tailing of full-length 16S rRNA and reverse transcription-PCR (RT-PCR) with domain-specific primers, and compared sequence libraries prepared from a marine sediment sample collected from the Yonaguni Knoll IV hydrothermal field.

## MATERIALS AND METHODS

### SAMPLING OF MARINE SEDIMENTS

The sediment samples used in this study were obtained from the Yonaguni Knoll IV hydrothermal field in the southern Okinawa Trough (24°50.544′N, 122°42.878′E, water depth: 1,371 m) using a push corer, and were collected during the JAMSTEC NT10-06 cruise involving *RV Natsushima* and *ROV Hyper-Dolphin* (Dive #1111, April 17, 2010). Sediment samples were anaerobically placed in autoclaved 250-ml glass bottles using a nitrogen flush and the bottles were sealed with a rubber cap and stored at 4°C until analysis.

### RNA EXTRACTION AND PURIFICATION

Bulk environmental RNA was extracted from 8 g of sediment using an RNA PowerSoil ^®^ Total RNA Isolation Kit (MO BIO Laboratories, Inc., Solana Beach, CA, USA) according to the manufacturer’s instructions. The extracted RNA was electrophoresed on a 2% agarose gel for 30 min in 1× TAE [Tris–acetate–ethylenediaminetetraacetic acid (EDTA)] buffer, and the gel was stained with 1× SYBR Green II (Life Technologies Japan, Tokyo, Japan) to visualize 16S and 23S rRNA. The rRNA was recovered from the gel using a Recochip (Takara Bio, Japan), and then further purified using a PureLink RNA Mini Kit (Life Technologies Japan) according to the manufacturer’s instructions. The quality of the recovered 16S rRNA was verified by electrophoresis using an automated capillary electrophoresis system (Experion; Bio-Rad Laboratories, Tokyo, Japan) and an Experion RNA HighSens Analysis Kit.

### POLY(A) TAILING AND COMPLEMENTARY DNA SYNTHESIS

**FIGURE 1:**
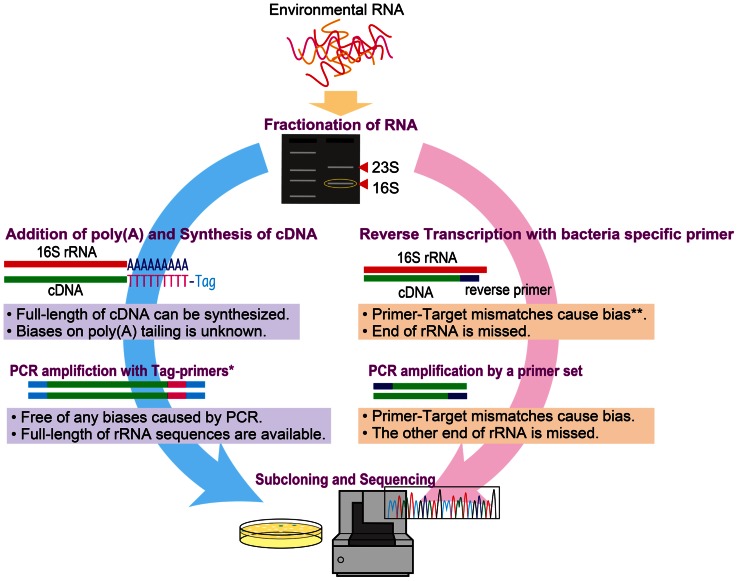
**Schematic illustrating the poly(A) tailing and RT-PCR methods for the study of active microbial communities.** *Tag primers target tagged sequences by adding an oligo dT for cDNA synthesis. **The bias at reverse transcription could be circumvented by using random hexamer primers instead of target-specific primers.

We compared two molecular approaches [poly(A) tailing and RT-PCR] for examining metabolically active microbial communities. The approaches are summarized in **Figure1**. Since the reaction buffer composition and source of poly(A) polymerase can reportedly affect the efficiency of the poly(A) tailing reaction (Raynal and Carpousis, 1999;Sillero et al., 2001), we used two commercially available poly(A) polymerases: *Escherichia coli* poly(A) polymerase (New England BioLabs, hereafter denoted as NEB) and Takara Bio poly(A) polymerase. Each poly(A) tailing reaction was conducted in 20 μl of reaction mixture containing 10 μl of purified 16S rRNA solution. The other components of the reaction mixture were as follows: for NEB polymerase, 1× reaction buffer (50 mM Tris–HCl, 250 mM NaCl, and 10 mM MgCl_2_), 1 mM ATP, and 0.25 U/μl of poly(A) polymerase; for Takara Bio, 1× reaction buffer [50 mM Tris–HCl, 10 mM MgCl_2_, 2.5 mM MnCl_2_, 250 mM NaCl, and 1 mM dithiothreitol (DTT)], 0.5 mg/ml of bovine serum albumin, 0.5 mM ATP, and 0.1 U/μl of poly(A) polymerase. After incubation at 37°C for 30 min, the reaction was stopped by adding 2 μl of 250 mM EDTA. The poly(A)-tailed 16S rRNA was subsequently purified using a NucleoSpin ^®^ RNA XS Kit (Takara Bio). The cDNA of full-length 16S rRNA was synthesized and amplified by PCR using a SMARTer^TM^ Pico PCR cDNA Synthesis Kit (Takara Bio) according to the manufacturer’s instructions.

### REVERSE TRANSCRIPTION-PCR

Reverse transcription-PCR was performed to obtain nearly full-length rRNA gene sequences from the purified 16S rRNA without poly(A) tailing using a One-Step PrimeScript RT-PCR Kit (Takara Bio). The bacterial domain-specific primers 26F (AGAGTTTGATCCTGGCTCA;Hicks et al., 1992) and 1492R (GGYTACCTTGTTACGACTT;Loy et al., 2002) were used for RT-PCR. The reaction mixture consisted of 1× PrimeScript buffer, 300 nM of each primer, 0.8 μl of PrimeScript Enzyme mix, 1 μl of 16S rRNA (diluted 1,000-fold), and water to 20 μl. First, reverse transcription was performed at 50°C for 30 min followed by inactivation of the reverse transcriptase at 94°C for 2.5 min. Next, synthesized cDNA was amplified by PCR under the following condition: 20 cycles of 94°C for 30 s, 54°C for 30 s, and 72°C for 90 s. The number of PCR cycles used in this study was determined by selecting a cycle number in the log-linear phase of the real-time PCR amplification curve (i.e., before the plateau phase). The PCR products were purified using NucleoSpin Extract II Columns (Takara Bio) and stored at -20°C until further analysis.

### CLONING AND SEQUENCING

The PCR products obtained using poly(A) tailing and RT-PCR were cloned into the pCR ^^®^^2.1-TOPO ^^®^^ vector and transformed into competent *E. coli* DH5α (Life Technologies Japan, Tokyo, Japan). For RT-PCR, the cloned inserts were sequenced using an ABI 3130xl genetic analyzer (Life Technologies Japan) with the primers M13M4, M13rev, 926R/F (Liu et al., 1997), and 1390R (Zheng et al., 1996). For the poly(A) tailing method, sequencing was first performed using the M13 primers followed by screening of the 16S rRNA sequence using a hidden Markov model implemented in version 3.0 of the HMMER software package (Eddy, 1998), as described elsewhere (Lagesen et al., 2007;Huang et al., 2009). The screened 16S rRNA inserts were sequenced using primers 338R/F (Amann et al., 1990), 515R/F (Walters et al., 2011), 926R/F, and 1390R to assemble full-length 16S rRNA sequences. A primer walking approach was employed for inserts that could not be sequenced using the primers described above. The sequences were trimmed and assembled to obtain consensus sequences using Sequencher software (Hitachi Software, Tokyo, Japan). Chimeric sequences were removed using the UCHIME program (Edgar et al., 2011) implemented in the Mothur Utility package (Schloss et al., 2009).

### DATA ANALYSIS

Alignment of all 16S rRNA sequences was performed using the ARB software package (Ludwig et al., 2004). Since some of the 16S rRNA sequences were fragmented after poly(A) tailing, a 600-bp fragment (corresponding to *E. coli* 16S rRNA positions 287–886) was used for comparisons of microbial diversity and community structure. Taxonomic assignments were made using Silva taxonomy and the Bayesian classifier. clustering sequences, calculation of diversity indices (i.e., Shannon and Simpson indices) and Libshuff test (Singleton et al., 2001;Schloss et al., 2004) were performed using the Mothur software package (Schloss et al., 2009;Hoshino et al., 2011). Phylogenetic tree was constructed by ARB software (Ludwig et al., 2004) using the neighbor-joining method (Saitou and Nei, 1987) with an Olsen correction. The coverage rate of the used primer set (26F-1492R) at the genus level was evaluated using TestPrime 1.0 program (Klindworth et al., 2013) using SILVA database SSU r114 with RefNR.

### NUCLEOTIDE SEQUENCE ACCESSION NUMBERS

All 16S rRNA sequences obtained in this study were deposited in the DDBJ/EMBL/GenBank nucleotide sequence databases under the accession numbers KC470861–KC471309.

## RESULTS AND DISCUSSION

### HMMER SCREENING OF 16S rRNA

Hmmer screening of 16S rRNA sequences obtained using the poly(A) tailing method resulted in the detection of 115 and 144 bacterial 16S rRNA sequences for the NEB and Takara poly(A) polymerase reactions, respectively. Approximately a half number of the total cDNA sequences (i.e., 107 and 92 sequences in the NEB and Takara cDNA libraries, respectively) were found to be 23S rRNA fragments according to the HMMER analysis. Interestingly, few cDNAs from mRNA were detected. The fragmented 23S rRNA sequences were excluded from the downstream analysis. Some fragmented 16S rRNA sequences were also observed in the poly(A) tailing libraries, suggesting that part of the 16S rRNA pool was damaged during the fractionation step by excision of the band and extraction of 16S rRNA from agarose gel.

Only one and three of the 16S rRNA sequences obtained using the NEB and Takara polymerases, respectively, were identified as archaeal 16S rRNA. This result was consistent with results from previous analyses of samples from the same location, which indicated that the archaeal population is generally smaller than the bacterial population (Yanagawa et al., 2012). In addition, a previous study of geothermally heated soil from Yellowstone National Park in the United States recovered no archaeal RNA sequences using the poly(A) tailing method, despite the fact that numerous archaeal 16S rRNA sequences were obtained using the PCR-based clone library method (Botero et al., 2005). Therefore, poly(A) tailing might have bias which underestimate archaeal population although it is unknown whether the low abundance of archaeal sequences in the poly(A) tailing libraries is from the native archaeal abundance or due to this bias. It is important to note here that RNA-based methods depend on the recovery of intracellular RNA; therefore, the results cannot be correlated with the cellular biomass or DNA copy number of the genomic pool.

### COMPARISON OF MICROBIAL DIVERSITY

**FIGURE 2:**
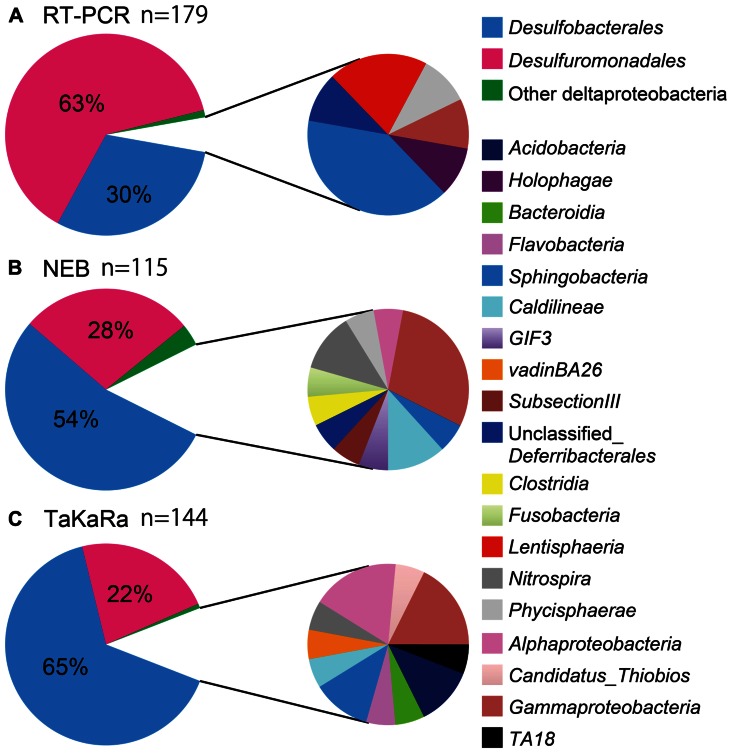
**The community structure of marine sediment microbial populations inferred from the results of RT-PCR and poly(A) tailing analyses**. *Deltaproteobacteria* (left) and other bacterial populations (right) were shown in pie charts. Chart **(A)** indicated microbial community obtained by RT-PCR, whereas chart **(B)** and chart **(C)** were by poly(A) tailing with NEB and Takara poly(A) polymerase, respectively.

More than 85% of the total 16S rRNA sequences obtained using the poly(A) tailing and conventional RT-PCR methods were found to be derived from the Deltaproteobacteria, indicating that sulfate-reducing bacteria are predominant members of sedimentary habitats (**Figure 2**, pie charts on the left). Conventional RT-PCR analysis identified 94% (169/179) of the sequences obtained as Deltaproteobacteria, whereas 85% (98/115) and 88% (127/144) of the bacterial 16S rRNA sequences obtained using the NEB and Takara polymerase poly(A) tailing methods, respectively, were identified as Deltaproteobacteria (**Figure 2**, pie charts on the left). Overall, these results are consistent with those of a previous RNA-based study of the same hydrothermal field (Yanagawa et al., 2012).

The Deltaproteobacteria orders Desulfuromonadales and Desulfobacterales, both of which contain sulfur- and/or sulfate-reducing bacteria, consistently appeared as predominant phylotypes in the clone libraries. However, there was a clear difference in the clonal frequency between libraries constructed using the two methods; the RT-PCR method indicated the predominance of Desulfuromonadales, while the poly(A)-tailing method indicated that Desulfobacterales predominate (**Figure 2**).

**FIGURE 3:**
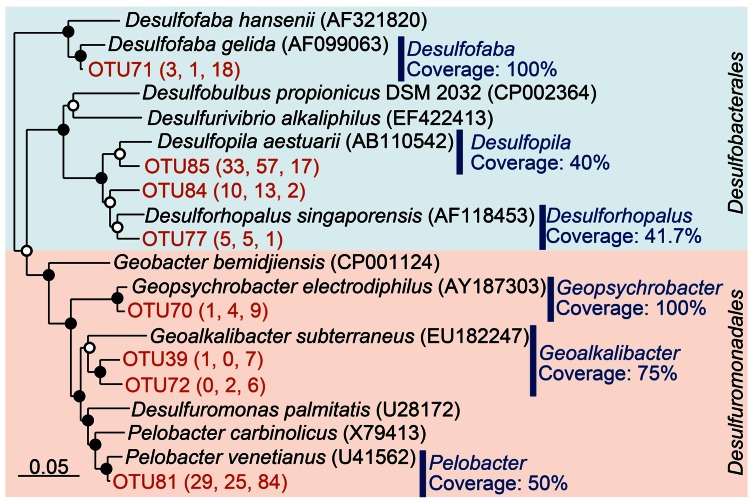
**Phylogenetic classification of *Deltaproteobacteria* sequences obtained in this study.** The tree was constructed by neighbor-joining analysis with an Olsen correction. Operational taxonomic units (OTUs) were defined as the clusters at 97% sequence identity and only OTUs containing more than five sequences were shown in the tree. The numbers in parenthesis indicate the number of clones obtained by NEB poly(A) polymerase, Takara poly(A) polymerase, and conventional RT-PCR, respectively (from left to right). Coverage of the primer set (26F-1492R) determined by TestPrime (http://www.arb-silva.de/search/testprime/) is shown for each genus. Bootstrap values are shown at branch nodes by closed circles (>80%) and open circles (<80%) as percentages of 1,000 replicates. Scale bar indicates 5% sequence divergence.

The detected sequences affiliated with Desulfuromonadales were mainly composed by the genera *Pelobacter*, *Geoalkalibacter*, and *Geopsychrobacter* (**Figure 3**). Almost half of the sequences from RT-PCR (84/179) and more than 25 sequences from both poly(A) libraries were classified to be *Pelobacter*, indicating predominance of this genus in the environment. TestPrime analysis (Klindworth et al., 2013) indicated that the coverage rates of the 26F and 1492R primers with perfect match for *Pelobacter*, *Geoalkalibacter*, and *Geopsychrobacter* are 50.0, 75.0, and 100%, respectively. On the other hand, the detected sequences of Desulfobacterales mainly consist of the genera *Desulfopila*, *Desulfofaba*, and *Desulforhopalus* (**Figure 3**), for which the coverage rates are 40, 100, and 41.7%, respectively. Among those three genera, the *Desulfopila*-related sequences were predominant in both poly(A) tailing clone libraries. The coverage rates of the detected genera within the Desulfobacterales were lower than those of Desulfuromonadales, resulting in lower abundance of Desulfobacterales in the RT-PCR libraries. Therefore, we infer that primer-dependent RT-PCR assay overestimated Desulfuromonadales but underestimated Desulfobacterales due to the primer bias.

Representatives of the Gammaproteobacteria and Sphingobacteria were relatively minor components of all three libraries we examined. Although some sequences derived from Lentisphaerae and Holophagae were only detected by RT-PCR, the poly(A) tailing libraries constructed using the two different polymerases revealed more diverse lineages than did the RT-PCR library. The clone libraries obtained from poly(A) tailing included some classes that were not detected by RT-PCR, such as *Nitrospira*, *Alphaproteobacteria*, and *Caldilineae*.

In theory, poly(A) tailing methods could also be used to obtain archaeal 16S rRNA, although a previous study failed to retrieve any archaeal 16S rRNA from geothermally heated soils from Yellowstone National Park (Botero et al., 2005). In this study, a total of four archaeal 16S rRNA sequences were obtained using the poly(A) tailing method.

**FIGURE 4:**
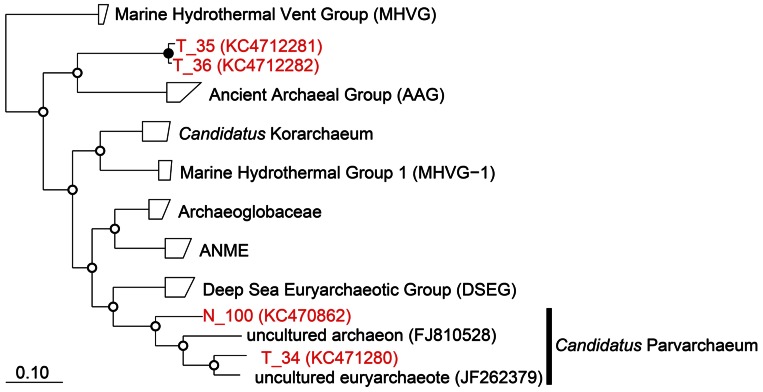
**Phylogenetic classification of archaeal 16S rRNA sequences obtained in this study.** The tree was constructed by neighbor-joining analysis with an Olsen correction. Bootstrap values were shown at branch point as percentages of 1,000 replicates. Bootstrap values are shown at branch nodes by closed circles (>80%) and open circles (<80%) as percentages of 1,000 replicates. Scale bar indicated 10% sequence divergence.

Two of these sequences were derived from *Candidatus* “Parvarchaeum” (Baker et al., 2010), which belonged to Deep-sea Hydrothermal Vent Euryarchaeotic Group (DHVEG-6;Takai and Horikoshi, 1999), while the other two sequences formed a new branch distinct from the ancient archaeal group (AAG;Takai and Horikoshi, 1999; **Figure 4**). Organisms belonging to DHVEG-6 are primarily associated with deep-sea hydrothermal vent systems (Takai and Horikoshi, 1999;Teske and Sørensen, 2008;Nunoura et al., 2012), but have also been found in marine sediment and anoxic soil. The AAG were first described as a hydrothermal vent lineage, and, consistent with the results of this study, were later found in the cold organic-rich subsurface environment (Sørensen and Teske, 2006). Due to primer mismatching, there have been few reports to date of the use of conventional PCR with published primer sets to detect the four archaeal 16S rRNA sequences we detected in this study. For example, all four sequences have one mismatch to A806F (Wang and Qian, 2009), while Arch958R (DeLong, 1992) has six mismatches to T_34 and N_100, and two mismatches to T_35 and T_36.

In addition, we retrieved 23S rRNA by poly(A) tailing with Takara polymerase: a total of 78 partial 23S rRNA sequences (~600 bp in length) were obtained. Although classification of the 23S rRNA sequences might be insufficient for the genus-level classification due to the limited number of 23S rRNA in the database, we found predominance of Deltaproteobacteria (60/78) containing Desulfobacterales (19/78), Desulfuromonadales (20/78), and unclassified sequences (19/78), consistently supporting our observation of 16S rRNA gene sequences.

### COMPARISON OF MICROBIAL COMMUNITY STRUCTURES

To compare the microbial community structures indicated by the poly(A) tailing and RT-PCR approaches, we calculated Shannon (*H*′) and Simpson diversity (1/*D*) indices for the 16S rRNA libraries. The highest diversity value was for the poly(A)-tailed sequences obtained using the NEB polymerase (**Table 1**). For the unique poly(A)-tailed sequences (i.e., singletons), the highest diversity indices were obtained using the Takara polymerase. In contrast, the RT-PCR method was associated with the lowest diversity indices, regardless of the similarity cutoff used or not (**Table 1**). The results of Libshuff analysis indicate that the two poly(A) clone libraries are statistically different from that of RT-PCR whereas poly(A) libraries are not significantly different (**Table 2**). Overall, these results indicate that the poly(A) tailing methods retrieve more diverse 16S rRNA sequences from the environment than does the conventional RT-PCR approach. In other words, it is important to recognize that primer-dependent molecular ecological approaches carry a risk of bias that could result in underestimation of microbial diversity. The bias effect may be more significant for microbial communities in rare and/or extreme habitats that have never been explored because we do not know exactly what organisms reside there.

**Table 1:** Diversity indices.

Similarity cutoff (%)	Shannon diversity index (*H*′)			Simpson diversity index (1/*D*)		
	RT-PCR	Poly(A) NEB	Poly(A) Takara	RT-PCR	Poly(A) NEB	Poly(A) Takara
0 (unique)	4.41	4.22	4.54	49.17	64.90	70.52
3	2.23	2.48	2.41	4.13	6.53	5.16

*NEB: New England BioLabs *Escherichia coli* poly(A) polymerase. Takara: Takara poly(A) polymerase.*

**Table 2:** *P*-values* estimating similarity among each treatment generated using Libshuff (10,000 randomizations) among the three clone libraries.

	*P*-value comparison of library (*Y*) with *X*a		
Library (*X*)	RT-PCR	NEB poly(A)	Takara poly(A)
RT-PCR	/	**0.0001**	**0.0003**
NEB poly(A)	**< 0.0001**	/	0.4009
Takara poly(A)	**0.0051**	0.2407	/

*P-values comparing either *X* to *Y* or *Y* to *X* indicate that the two communities are significantly different bold face values indicated significant difference (*P* < 0.0085, employing the Bonferroni correction).

## CONCLUSION AND PERSPECTIVES

For decades, PCR-mediated molecular ecological approaches have been used to investigate the diversity of microbial communities in a variety of natural habitats. The primer sequences for amplifying 16S rRNA (or its gene fragments) are based on known sequences contained in databases, targeting conserved regions that cover specific taxonomic groups. In this context, a critical issue in microbial ecology is the possibility of bias caused by mismatches between the published primers and the target sequences, especially for unidentified constituents of microbial communities in natural habitats. Bias of this sort has caused significant differences in estimates of microbial diversity and community structure, and also increases the difficulty of detecting previously unidentified organisms in the environment.

The poly(A) tailing of environmental 16S rRNA is totally independent of published PCR primers. In this study, we clearly showed that the poly(A) tailing approach holds potential for understanding of naturally occurring active microbial communities. This approach also has great potential for facilitating the discovery of as yet unknown microbes for which their 16S rRNA gene sequence do not match published primer sequences, although the potential bias of poly(A) tailing to rRNA genes needs to be studied further. By combining this approach with “deep sequencing” NGS technologies that allow for sequencing full-length 16S rRNAs, it may be possible in the future to obtain a detailed view of the true structure of microbial communities in natural habitats.

## Conflict of Interest Statement

The authors declare that the research was conducted in the absence of any commercial or financial relationships that could be construed as a potential conflict of interest.

## References

[B1] Acinas S. G. Sarma-Rupavtarm R. Klepac-Ceraj V. Polz M. F.(2005) PCR-induced sequence artifacts and bias: insights from comparison of two 16S rRNA clone libraries constructed from the same sample. *Appl. Environ. Microbiol.* 71 8966–8969 10.1128/AEM.71.12.8966-8969.2005 16332901 PMC1317340

[B2] Amann R. I. Binder B. J. Olson R. J. Chisholm S. W. Devereux R. Stahl D. A. (1990) Combination of 16S rRNA-targeted oligonucleotide probes with flow cytometry for analyzing mixed microbial populations. *Appl. Environ. Microbiol.* 56 1919–1925 2200342 10.1128/aem.56.6.1919-1925.1990 PMC184531

[B3] Baker B. J. Comolli L. R. Dick G. J. Hauser L. J. Hyatt D. Dill B. D. (2010) Enigmatic, ultrasmall, uncultivated archaea. *Proc. Natl. Acad. Sci. U.S.A.* 107 8806–8811 10.1073/pnas.0914470107 20421484 PMC2889320

[B4] Baker B. J. Tyson G. W. Webb R. I. Flanagan J. Hugenholtz P. Allen E. E. (2006) Lineages of acidophilic archaea revealed by community genomic analysis. *Science* 314 1933–1935 10.1126/science.1132690 17185602

[B5] Baker G. C. Smith J. J. Cowan D. A.(2003) Review and re-analysis of domain-specific 16S primers. *J. Microbiol. Methods* 55 541–555 10.1016/j.mimet.2003.08.009 14607398

[B6] Ben-Dov E. Shapiro O. H. Kushmaro A.(2012) ‘Next-base’ effect on PCR amplification. *Environ. Microbiol. Rep.* 4 183–188 10.1111/j.1758-2229.2011.00318.x 23757271

[B7] Botero L. M. D’Imperio S. Burr M. Mcdermott T. R. Young M. Hassett D. J. (2005) Poly(A) polymerase modification and reverse transcriptase PCR amplification of environmental RNA. *Appl. Environ. Microbiol.* 71 1267–1275 10.1128/AEM.71.3.1267-1275.2005 15746328 PMC1065135

[B8] Bru D. Martin-Laurent F. Philippot L. (2008) Quantification of the detrimental effect of a single primer-template mismatch by real-time PCR using the 16S rRNA gene as an example. *Appl. Environ. Microbiol.* 74 1660–1663 10.1128/AEM.02403-07 18192413 PMC2258636

[B9] DeLong E. F. (1992) Archaea in coastal marine environments. *Proc. Natl. Acad. Sci. U.S.A.* 89 5685–5689 10.1073/pnas.89.12.5685 1608980 PMC49357

[B10] Eddy S. R. (1998) Profile hidden Markov models. *Bioinformatics* 14 755–763 10.1093/bioinformatics/14.9.755 9918945

[B11] Edgar R. C. Haas B. J. Clemente J. C. Quince C. Knight R. (2011) UCHIME improves sensitivity and speed of chimera detection. *Bioinformatics* 27 2194–2200 10.1093/bioinformatics/btr381 21700674 PMC3150044

[B12] Fuhrman J. A. (2009) Microbial community structure and its functional implications. *Nature* 459 193–199 10.1038/nature08058 19444205

[B13] Hicks R. E. Amann R. I. Stahl D. A. (1992) Dual staining of natural bacterioplankton with 4′,6-diamidino-2-phenylindole and fluorescent oligonucleotide probes targeting kingdom-level 16S rRNA sequences. *Appl. Environ. Microbiol.* 58 2158–2163 1379029 10.1128/aem.58.7.2158-2163.1992 PMC195749

[B14] Hong S. Bunge J. Leslin C. Jeon S. Epstein S. S. (2009) Polymerase chain reaction primers miss half of rRNA microbial diversity. *ISME J.* 3 1365–1373 10.1038/ismej.2009.89 19693101

[B15] Hoshino T. Morono Y. Terada T. Imachi H. Ferdelman T. G. Inagaki F. (2011) Comparative study of subseafloor microbial community structures in deeply buried coral fossils and sediment matrices from the challenger mound in the porcupine seabight. *Front. Microbiol* 2: 231 10.3389/fmicb.2011.00231 PMC3218302 22110470

[B16] Huang Y. Gilna P. Li W. (2009) Identification of ribosomal RNA genes in metagenomic fragments. *Bioinformatics* 25 1338–1340 10.1093/bioinformatics/btp161 19346323 PMC2677747

[B17] Inagaki F. Sakihama Y. Inoue A. Kato C. Horikoshi K. (2002) Molecular phylogenetic analyses of reverse-transcribed bacterial rRNA obtained from deep-sea cold seep sediments. *Environ. Microbiol.* 4 277–286 10.1046/j.1462-2920.2002.00294.x 12030853

[B18] Klindworth A. Pruesse E. Schweer T. Peplies J. Quast C. Horn M. (2013) Evaluation of general 16S ribosomal RNA gene PCR primers for classical and next-generation sequencing-based diversity studies. *Nucleic Acids Res.* 41 e1 10.1093/nar/gks808 PMC3592464 22933715

[B19] Lagesen K. Hallin P. Rodland E. A. Staerfeldt H. H. Rognes T. Ussery D. W. (2007) RNAmmer: consistent and rapid annotation of ribosomal RNA genes. *Nucleic Acids Res.* 35 3100–3108 10.1093/nar/gkm160 17452365 PMC1888812

[B20] Liu W. T. Marsh T. L. Cheng H. Forney L. J. (1997) Characterization of microbial diversity by determining terminal restriction fragment length polymorphisms of genes encoding 16S rRNA. *Appl. Environ. Microbiol.* 63 4516–4522 9361437 10.1128/aem.63.11.4516-4522.1997 PMC168770

[B21] Loy A. Lehner A. Lee N. Adamczyk J. Meier H. Ernst J. (2002) Oligonucleotide microarray for 16S rRNA gene-based detection of all recognized lineages of sulfate-reducing prokaryotes in the environment. *Appl. Environ. Microbiol.* 68 5064–5081 10.1128/AEM.68.10.5064-5081.2002 12324358 PMC126405

[B22] Ludwig W. Strunk O. Westram R. Richter L. Meier H. Yadhukumar (2004) ARB: a software environment for sequence data. *Nucleic Acids Res.* 32 1363–1371 10.1093/nar/gkh293 14985472 PMC390282

[B23] Nunoura T. Takaki Y. Kazama H. Hirai M. Ashi J. Imachi H. (2012) Microbial diversity in deep-sea methane seep sediments presented by SSU rRNA gene tag sequencing. *Microbes Environ.* 27 382–390 10.1264/jsme2.ME12032 22510646 PMC4103545

[B24] Raynal L. C. Carpousis A. J. (1999) Poly(A) polymerase I of *Escherichia coli*: characterization of the catalytic domain, an RNA binding site and regions for the interaction with proteins involved in mRNA degradation. *Mol. Microbiol.* 32 765–775 10.1046/j.1365-2958.1999.01394.x 10361280

[B25] Saitou N. Nei M. (1987) The neighbor-joining method: a new method for reconstructing phylogenetic trees. *Mol. Biol. Evol.* 4 406–425 3447015 10.1093/oxfordjournals.molbev.a040454

[B26] Schloss P.D. Larget B.R. Handelsman J. (2004) Integration of microbial ecology and statistics: a test to compare gene libraries. *Appl. Environ. Microbiol.* 70 5485–5492 10.1128/AEM.70.9.5485-5492.2004 15345436 PMC520927

[B27] Schloss P. D. Westcott S. L. Ryabin T. Hall J. R. Hartmann M. Hollister E. B. (2009) Introducing mothur: open-source, platform-independent, community-supported software for describing and comparing microbial communities. *Appl. Environ. Microbiol.* 75 7537–7541 10.1128/AEM.01541-09 19801464 PMC2786419

[B28] Sillero M. A. Socorro S. Baptista M. J. Del Valle M. De Diego A. Sillero A. (2001) Poly(A) polymerase from *Escherichia coli* adenylylates the 3′-hydroxyl residue of nucleosides, nucleoside 5′-phosphates and nucleoside(5′)oli-gophospho(5′)nucleosides (NpnN). *Eur. J. Biochem.* 268 3605–3611 10.1046/j.1432-1327.2001.02271.x 11422392

[B29] Singleton D. R. Furlong M. A. Rathbun S. L. Whitman W. B. (2001) Quantitative comparisons of 16S rRNA gene sequence libraries from environmental samples. *Appl. Environ. Microbiol.* 67 4374–4376 10.1128/AEM.67.9.4374-4376.2001 11526051 PMC93175

[B30] Sipos R. Szekely A. J. Palatinszky M. Revesz S. Marialigeti K. Nikolausz M. (2007) Effect of primer mismatch, annealing temperature and PCR cycle number on 16S rRNA gene-targetting bacterial community analysis. *FEMS Microbiol. Ecol.* 60 341–350 10.1111/j.1574-6941.2007.00283.x 17343679

[B31] Sogin M. L. Morrison H. G. Huber J. A. Welch D. M. Huse S. M. Neal P. R. (2006) Microbial diversity in the deep sea and the underexplored “rare biosphere”. *Proc. Natl. Acad. Sci. U.S.A.* 103 12115–12120 10.1073/pnas.0605127103 16880384 PMC1524930

[B32] Sørensen K. B. Teske A. (2006) Stratified communities of active Archaea in deep marine subsurface sediments. *Appl. Environ. Microbiol*. 72 4596–4603 10.1128/AEM.00562-06 16820449 PMC1489303

[B33] Takai K. Horikoshi K. (1999) Genetic diversity of archaea in deep-sea hydrothermal vent environments. *Genetics* 152 1285–1297 10430559 10.1093/genetics/152.4.1285 PMC1460697

[B34] Takami H. Noguchi H. Takaki Y. Uchiyama I. Toyoda A. Nishi S. (2012) A deeply branching thermophilic bacterium with an ancient acetyl-CoA pathway dominates a subsurface ecosystem. *PLoS ONE* 7: e30559 10.1371/journal.pone.0030559 PMC3267732 22303444

[B35] Teske A. Sørensen K. B. (2008) Uncultured archaea in deep marine subsurface sediments: have we caught them all? *ISME J.* 2 3–18 18180743 10.1038/ismej.2007.90

[B36] Vergin K. L. Urbach E. Stein J. L. Delong E. F. Lanoil B. D. Giovannoni S. J. (1998) Screening of a fosmid library of marine environmental genomic DNA fragments reveals four clones related to members of the order Planctomycetales. *Appl. Environ. Microbiol.* 64 3075–3078 9687477 10.1128/aem.64.8.3075-3078.1998 PMC106819

[B37] Walters W. A. Caporaso J. G. Lauber C. L. Berg-Lyons D. Fierer N. Knight R. (2011) PrimerProspector: de novo design and taxonomic analysis of barcoded polymerase chain reaction primers. *Bioinformatics* 27 1159–1161 10.1093/bioinformatics/btr087 21349862 PMC3072552

[B38] Wang Y. Qian P. Y. (2009) Conservative fragments in bacterial 16S rRNA genes and primer design for 16S ribosomal DNA amplicons in metagenomic studies. *PLoS ONE* 4: e7401 10.1371/journal.pone.0007401 PMC2754607 19816594

[B39] Webster N. S. Taylor M. W. Behnam F. Lucker S. Rattei T. Whalan S. (2010) Deep sequencing reveals exceptional diversity and modes of transmission for bacterial sponge symbionts. *Environ. Microbiol.* 12 2070–2082 21966903 10.1111/j.1462-2920.2009.02065.x PMC2936111

[B40] Yanagawa K. Morono Y. De Beer D. Haeckel M. Sunamura M. Futagami T. (2012) Metabolically active microbial communities in marine sediment under high-CO2 and low-pH extremes. *ISME J.* 7 555–567 10.1038/ismej.2012.124 23096400 PMC3578575

[B41] Zheng D. Alm E. W. Stahl D. A. Raskin L. (1996) Characterization of universal small-subunit rRNA hybridization probes for quantitative molecular microbial ecology studies. *Appl. Environ. Microbiol.* 62 4504–4513 8953722 10.1128/aem.62.12.4504-4513.1996 PMC168277

